# Clinical Factors Associated With Physician Referral During Ambulatory Visits to Non-psychiatric Physicians: A National Analysis of National Ambulatory Medical Care Survey (NAMCS) 2010–2015

**DOI:** 10.7759/cureus.106618

**Published:** 2026-04-07

**Authors:** Michael U Mochu, Akinyele Oladimeji, Esther K Otu, Abiodun Adegbite, Celestine I Ogwu, Ikenna C Madubuike

**Affiliations:** 1 Family Medicine, University of Nigeria College of Medicine, Enugu, NGA; 2 Family Medicine, Alberta Health Services, Edmonton, CAN; 3 Internal Medicine/Neurology, Washington University of Health and Science, San Pedro, BLZ; 4 Medicine and Surgery, University of Ibadan, Oyo, NGA; 5 Internal Medicine, Richmond Gabriel University, Kingstown, VCT; 6 Operations, Favored Healthcare Services, Buford, USA; 7 Carey Business School, Johns Hopkins University, Baltimore, USA

**Keywords:** ambulatory care, depression, health disparities, namcs, primary care, psychiatric referral

## Abstract

Background: Mental health care is increasingly delivered in primary care settings in the United States, yet referral patterns from non-psychiatric physicians to other providers remain incompletely described.

Objective: The objective of this study is to examine demographic and clinical factors associated with referral to another physician during adult ambulatory visits to non-psychiatric physicians.

Methods: A cross-sectional secondary analysis was conducted using the National Ambulatory Medical Care Survey from 2010 through 2015. The study included visits by adults aged 18 years and older to non-psychiatric physicians. Depression was identified using the survey indicator for current depression. The primary outcome was referral to another physician during the visit. Survey weighted logistic regression accounting for survey settings was utilized.

Results: The final sample included 150,287 visits, representing 3.86 billion weighted visits nationally. In unadjusted analysis, depression was associated with higher odds of referral (OR = 1.31; 95% CI: 1.17-1.47; p < 0.001). In adjusted analysis, this association was reduced and remained non-significant (aOR = 1.13; 95% CI: 1.00-1.27; p = 0.051). Greater chronic disease burden and racial and ethnic minority status were independently associated with referral.

Conclusion: Referral during adult ambulatory visits reflects patient complexity and demographic factors, while depression alone was not independently associated with referral after adjustment.

## Introduction

The prevalence of mental disorders is high in the United States, posing a substantial burden on the healthcare system, as reflected in rising medical expenditures and its association with reduced life expectancy, with mental health care utilization increasing from 7.0% in 1999 to 13.1% in 2018 [1]. Conditions such as depression, anxiety disorders, bipolar disorder, and substance use disorders are very widespread and are often associated with chronic medical conditions that contribute to prolonged functional impairment and increased healthcare utilization [2]. Though there are numerous reasons as to why the mentally ill may fail to receive treatment (e.g., stigma, lack of them seeing any need to receive treatment), affordability and other insurance-related aspects are one of the causes of this treatment gap [3,4]. Promotion, prevention, and early intervention strategies may produce the greatest impact on people’s health and well-being [5].

Considering the transformations of policy and care delivery, it is essential to prepare the primary care providers to deal with mental health requirements [6]. It would seem that a general practitioner is well set to identify mental disorders in patients presenting with physical complaints [7]. Anxious and depressed patients are more than two times more likely to have multiple unexplained somatic symptoms than non-anxious and non-depressed patients [8]. Therefore, the role played by the non-psychiatric doctor is significant in detecting psychiatric symptoms, initial treatment, and the determination of when the client would be referred to specialized mental health services [9].

Mental health practitioners make significant choices in mental care continuum in terms of ambulatory visits [10]. In combination with specialized mental health care, mental health care is usually offered by other primary health and social services [11]. Conversely, the lack of or delayed referrals may lead to additional symptom progression, increased health spending, emergency department (ED) visits, and other adverse effects, including hospitalization and the risk of suicide [12]. Factors influencing referrals by general practitioners to mental health services include disease characteristics, patient factors (such as age, gender, and presentation of psychological symptoms), and healthcare system factors, including limited access to specialists, long travel distances or wait times, and challenges in communication and care coordination [13]. They might be demographic variables (age, sex, race, and ethnicity) or insurance coverage, presenting diagnosis, comorbid medical conditions, psychiatric symptoms severity, time constraints during visits, access to mental health providers, and perceived stigma [14].

The disparity in mental health access remains a permanent feature in the United States [15]. It is pointed out that fewer racial and ethnic minorities, uninsured individuals, and areas with under-service are most likely to receive specialty mental health care [16]. To define potential gaps in care and inform the strategies to promote equal access to mental health care, there is therefore an urgent need to understand demographic and clinical factors that are associated with a psychiatric referral of patients in clinical ambulatory settings in practice [17,18].

Depression is a common mental disorder characterized by persistent low mood, loss of interest or pleasure, and a range of cognitive and somatic symptoms that impair daily functioning. Clinical diagnosis is typically based on established criteria requiring the presence of core symptoms, such as depressed mood or anhedonia, along with additional features including changes in sleep, appetite, energy, concentration, or feelings of guilt, persisting for a defined duration and causing functional impairment. Depression frequently co-occurs with chronic medical conditions and contributes to increased healthcare utilization and complexity of care [1,2].

The analysis of the study is based on the National Ambulatory Medical Care Survey (NAMCS). NAMCS is a survey that is nationally representative of outpatient visits to office practicing physicians in the United States and it has immense data on patient demographicsf., diagnosis, services provided and visit characteristics [19]. The patterns of care, including referral practice, in the normal healthcare delivery system may be analyzed using NAMCS through the acquisition of visit-based data across different geographical regions and clinical contexts [20].

The objective of the study is to identify demographic and clinical factors associated with referral to another physician during ambulatory visits to non-psychiatric physicians in the United States. The study findings will give insights into the existing trends in referrals via ambulatory care and may guide specific interventions, clinical practice, and policy interventions aimed at enhancing early identification, proper referral, and equal access to mental health care in the entire country.

## Materials and methods

Study design and data source

This study utilized a cross-sectional secondary analysis of nationally representative ambulatory visit data from the NAMCS for the years 2010-2015 [21]. The NAMCS is conducted annually by the National Center for Health Statistics and uses a multistage probability sampling design to produce national estimates of visits to nonfederal office-based physicians in the United States. The survey collects information on patient demographics, clinical conditions, services provided, and visit characteristics. Public use microdata files for each study year were appended to create a pooled dataset. Survey design variables including patient visit weights, primary sampling units, and strata, were retained to permit weighted national inference.

Study population

This study focused only on the ambulatory visits of adult patients ≥ 18 years. All psychiatrists' visits were excluded to concentrate only on non-psychiatric physicians. Physician specialty was identified based on specialty variables. Also, this study dropped all visits coded as psychiatry prior to analysis. Furthermore, the observations that reported missing physician specialty were excluded to ensure that the study population consisted only of clearly identified non-psychiatric physician visits. After these restrictions, the final analytic sample consisted of 150,287 unweighted visits, representing an estimated 3.86 billion ambulatory visits during the study period.

Variables and measures

The primary outcome was referral to another physician during the visit, defined using the NAMCS survey variable “referred to other physician” (REFOTHMD), which indicates whether the patient was referred to a different physician. This variable captures referral to a physician of any specialty and does not distinguish psychiatric referrals. This variable was coded as a binary outcome. The primary exposure was current depression status, defined using the survey indicator “depression present” (DEPRN), which reflects physician-documented depression at the time of the visit. This measure is not based on a standardized diagnostic questionnaire or uniform diagnostic criteria within the survey but rather on clinical documentation as recorded in NAMCS [21]. Depression was coded as a binary variable. Covariates were selected based on clinical relevance and prior literature examining referral patterns. Age was treated as a continuous variable in years. Sex was categorized as male or female. Race and ethnicity were classified using the survey race ethnicity variable with non-Hispanic White as the reference group. Chronic disease burden was measured using the total number of chronic conditions (TOTCHRON) recorded for the visit and was modeled as a continuous variable. Metropolitan status (MSA) was categorized as metropolitan statistical area or non-metropolitan area. All variables were defined directly from the public use dataset without transformation beyond recoding into analysis categories as described.

Missing data

Missingness of the data was examined for all the variables included in the study. However, after restrictions to adult patients and exclusion of visits with missing physician specialty, no missing data was reported for both the outcome, exposure, and covariates. Therefore, this study utilized complete case analysis.

Statistical analysis

The analysis in this study accounted for the complex survey design of NAMCS using masked sampling stratum (CSTRATM), provider marker (CPSUM) and patient visit weight (PATWT) as provided by the survey. Descriptive statistics was conducted for the study population and grouped by referral status. For the group comparisons, both the survey design t-test and chi-square test were applied for continuous and categorical variables, respectively. Unadjusted associations between depression and referral were first evaluated using survey-weighted logistic regression. A multivariable survey weighted logistic regression model was then constructed to estimate adjusted odds ratios for referral, including age, sex, race and ethnicity, depression status, total chronic conditions, and metropolitan status. Adjusted odds ratios with 95 percent confidence intervals were reported. A multicollinearity test was conducted, and the findings revealed no multicollinearity issue since the variance inflation factor (VIF) ranged from 1.01 to 1.15 with a mean VIF of 1.07. In addition, normality of continuous variables was assessed using the Shapiro-Wilk test, and all variables demonstrated p-values greater than 0.05, supporting the assumption of normality for the application of parametric tests. All data management and statistical analyses were conducted using Stata version 18 (StataCorp LLC, College Station, Texas, USA), and a two-sided p-value of less than 0.05 was considered statistically significant.

Ethical considerations

The study used publicly available, deidentified data from the NAMCS. The dataset contains no direct patient identifiers and is intended for public use research. Therefore, institutional review board approval was not required.

## Results

Table 1 presents baseline characteristics of adult ambulatory visits by referral status.

**Table 1:** Weighted Characteristics of Adult Ambulatory Visits by Referral Status, NAMCS 2010 to 2015 Values represent weighted national estimates from the National Ambulatory Medical Care Survey and correspond to 150,287 unweighted visits, representing 3,858,492,517 weighted visits nationally. Percentages are row percentages within each category. Continuous variables were compared using survey-adjusted t-tests. Categorical variables were compared using survey-adjusted chi-square tests converted to design-based F statistics. MSA, Metropolitan Statistical Area; -, Intentionally Left Blank; NAMCS, National Ambulatory Medical Care Survey The table was generated by the authors using Stata version 18 [21].

Characteristic	Referral (N = 354,999,628)	No Referral (N = 3,503,492,889)	Test Statistic	p-value
Patient age in years (mean ± SD)	55.60 ± 16.88	54.85 ± 18.58	t = -1.91	0.056
Total chronic conditions (mean ± SD)	1.77 ± 1.98	1.27 ± 2.10	t = -9.37	<0.001
Sex, n (%)	-	-	F = 7.84	0.005
Male	206,412,285 (9%)	2,149,893,538 (91%)	-	-
Female	148,587,342 (10%)	1,353,599,351 (90%)	-	-
Depression, n (%)	-	-	F = 21.20	<0.001
No depression	303,382,367 (9%)	3,101,276,697 (91%)	-	-
Depression	51,617,261 (11%)	402,216,192 (89%)	-	-
Race/Ethnicity, n (%)	-	-	F = 6.60	<0.001
Non-Hispanic White	240,283,878 (9%)	2,557,598,418 (91%)	-	-
Non-Hispanic Black	46,990,411 (11%)	371,393,128 (89%)	-	-
Hispanic	45,576,668 (11%)	384,395,304 (89%)	-	-
Non-Hispanic Other	22,148,670 (10%)	190,106,039 (90%)	-	-
Metropolitan Status, n (%)	-	-	F = 0.06	0.811
MSA	322,517,674 (9%)	3,175,142,674 (91%)	-	-
Non-MSA	32,481,954 (9%)	328,350,216 (91%)	-	-

The results indicate that 354,999,628(9%) of weighted ambulatory visits involved referral to another physician, while 3,503,492,889(91%) weighted visits did not involve referral. The mean age was similar between groups, 55.60(16.88) years among referred visits and 54.85 (18.58) years among non-referred visits, and this difference did not reach statistical significance, p = 0.056. The mean number of chronic conditions was higher among referred visits, 1.77 (1.98), compared with 1.27 (2.10) among non-referred visits, and this difference was statistically significant, p < 0.001. Sex distribution differed by referral status, p = 0.005, with 206,412,285(9%) of male visits and 148,587,342(10 percent) of female visits resulting in referral. Depression status was also associated with referral, p < 0.001. Among visits involving patients without depression, 303,382,367(9%) resulted in referral, whereas among visits involving patients with depression, 51,617,261(11%) resulted in referral. Race and ethnicity differed significantly by referral status, p<0.001. Referral occurred in 240,283,878(9%) of non-Hispanic White visits, 46,990,411(11%) of non-Hispanic Black visits, 45,576,668(11%) of Hispanic visits, and 22,148,670(10%) of non-Hispanic Other visits. Metropolitan status was not associated with referral, p = 0.811, with similar referral proportions observed in MSA and non-MSA locations.

**Figure 1:**
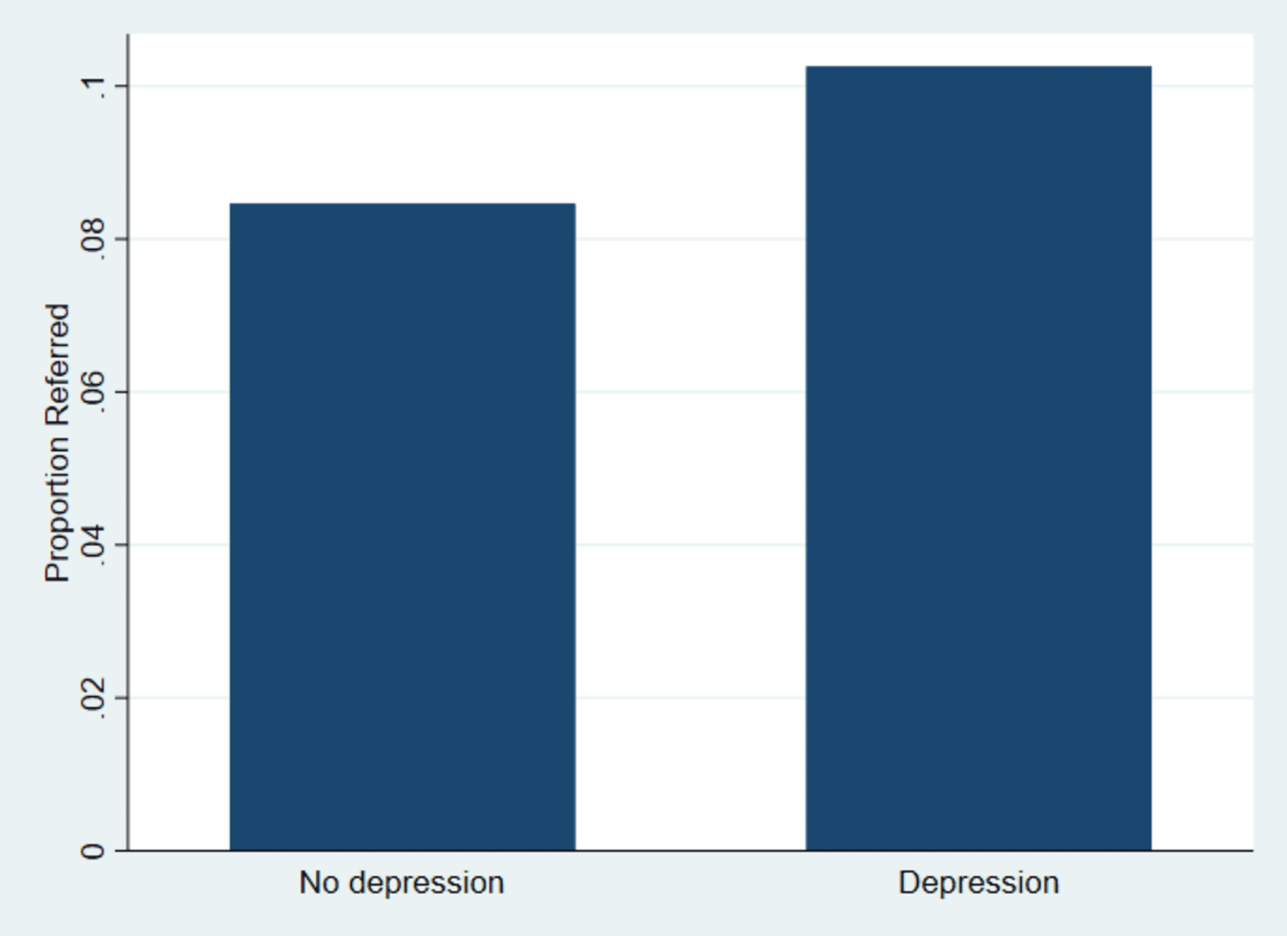
Weighted Proportion of Visits with Referral by Depression Status

Figure 1 illustrates the weighted proportion of ambulatory visits resulting in referral to another physician according to depression status.

The figure shows that patients presenting with depression are more readily identified as needing specialized mental health care, leading to higher referral rates.

Table 2 presents the unadjusted association between depression and referral to another physician among adult ambulatory visits.

**Table 2:** Survey-Weighted Logistic Regression for Unadjusted Association between Depression and Referral Odds ratios were estimated using survey weighted logistic regression accounting for the complex sampling design of the National Ambulatory Medical Care. CI denotes confidence interval. The table was generated by authors using Stata version 18 [21].

Variable	Odds Ratio	95% CI	p-value
Depression	-	-	-
Depression vs No Depression	1.31	1.17–1.47	<0.001

The results indicate that referral occurred in a higher proportion of visits among patients with depression compared with those without depression. The weighted referral proportion was approximately 0.10 among visits involving patients with depression and approximately 0.08 to 0.09 among visits involving patients without depression. This pattern is consistent with the unadjusted logistic regression findings in Table 2 showing higher odds of referral among visits with depression.

In unadjusted analysis, visits involving patients with depression had higher odds of referral compared with visits involving patients without depression. The odds ratio was 1.31 with a 95 percent confidence interval of 1.17 to 1.47, and this association was statistically significant, p < 0.001. These findings indicate that, prior to adjustment for demographic and clinical factors, depression was associated with greater likelihood of referral during the visit.

Table 3 presents the multivariable survey-weighted logistic regression analysis examining factors independently associated with referral to another physician among adult ambulatory visits.

**Table 3:** Survey-Weighted Multivariable Logistic Regression for Adjusted Association between Clinical Factors and Referral Adjusted OR, Adjusted Odds Ratio; CI, Confidence Interval; Std Error, Standard Error; t, t-statistic; MSA, Metropolitan Statistical Area.

Variable	Adjusted OR	95% CI	Std Error	t	p-value
Age, per year	0.997	0.995–0.999	0.001	-2.40	0.017
Female vs Male	1.13	1.03–1.25	0.055	2.59	0.010
Race/Ethnicity					
Non-Hispanic Black vs Non-Hispanic White	1.31	1.12–1.53	0.104	3.45	0.001
Hispanic vs Non-Hispanic White	1.27	1.07–1.51	0.112	2.76	0.006
Non-Hispanic Other vs Non-Hispanic White	1.27	1.03–1.56	0.133	2.25	0.024
Depression vs No Depression	1.13	1.00–1.27	0.069	1.95	0.051
Total chronic conditions	1.14	1.11–1.18	0.016	9.38	<0.001
Non MSA vs MSA	1.01	0.81–1.25	0.111	0.07	0.947

In the adjusted model, the findings revealed that age was modestly associated with referral, with each additional year associated with slightly lower odds of referral, adjusted odds ratio 0.997, 95 percent confidence interval 0.995 to 0.999, p = 0.017. Female visits had higher odds of referral compared with male visits, adjusted odds ratio 1.13, 95 percent confidence interval 1.03 to 1.25, p = 0.010. Race and ethnicity were significantly associated with referral. Compared with non-Hispanic White visits, higher odds of referral were observed among non-Hispanic Black visits, adjusted odds ratio 1.31, 95 percent confidence interval 1.12 to 1.53, p = 0.001; Hispanic visits, adjusted odds ratio 1.27, 95 percent confidence interval 1.07 to 1.51, p = 0.006; and non-Hispanic Other visits, adjusted odds ratio 1.27, 95 percent confidence interval 1.03 to 1.56, p = 0.024. Depression was associated with higher odds of referral, adjusted odds ratio 1.13, 95 percent confidence interval 1.00 to 1.27, but this association was not statistically significant, p = 0.051. Total chronic conditions were strongly associated with referral, with each additional chronic condition associated with higher odds of referral, adjusted odds ratio 1.14, 95 percent confidence interval 1.11 to 1.18, p < 0.001. Metropolitan status was not associated with referral, adjusted odds ratio 1.01, 95 percent confidence interval 0.81 to 1.25, p = 0.947.

## Discussion

This study examined demographic and clinical factors associated with referral to another physician during adult ambulatory visits to non-psychiatric physicians in the United States. In unadjusted analysis, visits involving patients with depression were associated with higher odds of referral. After adjustment for age, sex, race and ethnicity, chronic disease burden, and metropolitan status, the association between depression and referral was reduced and was not statistically significant at 5% level of significance. In contrast, greater chronic disease burden and racial and ethnic minority status were independently associated with higher odds of referral. Age showed a small inverse association with referral, and metropolitan status was not associated with referral after adjustment.

These findings should be considered in the context of changes in mental health care delivery within primary care settings. Prior work has shown that primary care physicians increasingly address mental health concerns during office visits [6,9]. As more mental health care is delivered within primary care, referral to specialty services may be influenced by the presence of additional medical complexity rather than depression alone. The higher odds of referral among visits with greater chronic disease burden are consistent with evidence that depression frequently co-occurs with multiple somatic conditions [8] and that patients presenting with physical complaints often have underlying depressive or anxiety disorders [7]. Medical complexity may prompt referral because of diagnostic uncertainty, need for subspecialty evaluation, or coordination of care across disciplines. The attenuation of the depression association after adjustment suggests that part of the unadjusted relationship reflects the broader burden of comorbidity rather than depression in isolation.

The observed differences by race and ethnicity align with documented disparities in mental health treatment patterns in the United States [16,17]. Studies have shown variation in mental health service use across racial and ethnic groups, influenced by access, availability of specialty services, and community-level supply factors [16,17]. In this study, non-Hispanic Black and Hispanic visits were associated with higher odds of referral compared with non-Hispanic White visits. This pattern may reflect differences in clinical presentation, communication patterns, or physician perceptions that influence referral decisions, as suggested in experimental work examining referral behavior for depressive symptoms [18]. It may also reflect differences in where mental health care is delivered, as primary care may serve as a key access point for populations with barriers to specialty care [14].

National data indicate that unmet need for mental health care remains common across insurance and demographic groups [3,4]. At the same time, mental health care utilization has increased over recent decades [1]. Referral from primary care to specialty mental health services represents one pathway through which patients may access additional care. The current findings suggest that referral decisions are not driven solely by documented depression status but are associated with broader clinical and demographic factors. This pattern is consistent with evidence that referral practices are shaped by physician judgment, patient complexity, and system-level constraints [10,18]. Physician clinical judgment plays a critical role in referral decisions during ambulatory visits, often extending beyond the presence of depression alone [18]. Referral decisions are frequently influenced by factors such as perceived diagnostic uncertainty, the severity of the patient's presentation, and the necessity for specialized evaluation or treatment that primary care providers may not be equipped to deliver [10,18]. Primary care physicians must weigh the complexity of each case, considering not only psychiatric symptoms but also comorbid medical conditions and psychosocial factors that may complicate diagnosis and management [9,13]. Interventions targeting referral processes, such as educational programs and quality indicators for referral pathways, have been proposed to improve coordination between primary and secondary mental health services [11,12]. Barriers to referral, including provider-level and system-level factors, have also been described in studies of primary care referral to psychotherapy services [13].

Current United States clinical guidance emphasizes screening and appropriate management of depression in primary care. The US Preventive Services Task Force (USPSTF) recommends screening for depression in the adult population when systems are in place to ensure accurate diagnosis, effective treatment, and appropriate follow-up [22]. Effective follow-up may include management within primary care or referral to specialty mental health services when indicated. The findings of this study suggest that referral patterns vary according to patient characteristics and comorbidity burden. Understanding how referral decisions align with recommended screening and management frameworks is important for ensuring that patients with depression receive timely and appropriate care.

Strengths and limitations of the study

This study has several strengths and limitations. The use of the NAMCS provides nationally representative data and allows examination of referral patterns across a large and diverse sample [19,20]. The survey design incorporates stratification, clustering, and sampling weights, which were accounted for in the analysis. However, the cross-sectional design limits the ability to assess temporal relationships between depression status and referral. Depression and chronic conditions were identified using survey documented indicators and may be subject to misclassification or reporting differences across practices. The analysis excluded variables with substantial structural missingness across survey years, which may limit examination of certain clinical factors not consistently captured in the dataset. Furthermore, the use of the REFOTHMD variable to define referral. This measure does not distinguish between types of providers, such as psychiatrists versus non-physician mental health professionals, and therefore limits the interpretation of referral pathways. Differences in access, cost, and wait times across provider types could not be assessed. These findings have important implications for clinical practice and health policy, suggesting that referral decisions in ambulatory care are influenced more by overall patient complexity than depression alone, highlighting the need for integrated care models and improved coordination between primary care and specialty services to optimize patient management. Future research should utilize longitudinal data to examine referral follow-through, timing, and patient outcomes, as well as the integration of mental health services within primary care settings. Additionally, studies incorporating provider-level factors, visit duration, and system-level constraints may help to better understand the drivers of referral decisions and variations in care pathways.

## Conclusions

This study highlights that referral to another physician during adult ambulatory visits is associated with patient demographic characteristics and comorbidity burden rather than depression status alone. Although depression was linked to referral in unadjusted analysis, this relationship was reduced and became non-significant after accounting for age, sex, race and ethnicity, and chronic conditions. Chronic disease burden and racial and ethnic minority status were independently associated with referral. These findings suggest that referral decisions in non-psychiatric settings may reflect broader clinical complexity and system level factors. Efforts to improve coordination between primary care and mental health services should consider how comorbidity and demographic factors shape referral patterns. Future research should examine referral quality, follow through, and longitudinal outcomes to better understand pathways to specialty mental health care.
